# Current progress in application of polymeric nanofibers to tissue engineering

**DOI:** 10.1186/s40580-019-0209-y

**Published:** 2019-11-08

**Authors:** Sorour Nemati, Se-jeong Kim, Young Min Shin, Heungsoo Shin

**Affiliations:** 1 Department of Bioengineering, Hanyang University, 222 Wangsimni-ro, Seongdong-gu, Seoul, 04763 Republic of Korea; 2 BK21 Plus Future Biopharmaceutical Human Resources Training and Research Team, 222 Wangsimni-ro, Seongdong-gu, Seoul, 04763 Republic of Korea; 3 Department of Nanobiomedical Science & BK21 PLUS NBM Global Research Center for Regenerative Medicine, Dankook University, Cheonan, 31116 Republic of Korea

**Keywords:** Tissue engineering, Extracellular matrix, Polymeric nanofibers, Electrospinning, Functional nanofibers, Tissue engineering applications

## Abstract

Tissue engineering uses a combination of cell biology, chemistry, and biomaterials to fabricate three dimensional (3D) tissues that mimic the architecture of extracellular matrix (ECM) comprising diverse interwoven nanofibrous structure. Among several methods for producing nanofibrous scaffolds, electrospinning has gained intense interest because it can make nanofibers with a porous structure and high specific surface area. The processing and solution parameters of electrospinning can considerably affect the assembly and structural morphology of the fabricated nanofibers. Electrospun nanofibers can be made from natural or synthetic polymers and blending them is a straightforward way to tune the functionality of the nanofibers. Furthermore, the electrospun nanofibers can be functionalized with various surface modification strategies. In this review, we highlight the latest achievements in fabricating electrospun nanofibers and describe various ways to modify the surface and structure of scaffolds to promote their functionality. We also summarize the application of advanced polymeric nanofibrous scaffolds in the regeneration of human bone, cartilage, vascular tissues, and tendons/ligaments.

## Introduction

The high occurrence of tissue injury and organ failure has caused the demand for organ transplantation to increase year by year [1]. Tissue engineering provides an alternative approach to the restoration of injured tissue while circumventing the drawbacks associated with autologous and allogeneic tissue transplantation [2]. To achieve the fabrication of three dimensional (3D) tissue, tissue engineering requires knowledge of cell biology, chemistry, materials science, nanotechnology, and micro- and nano-fabrication [3]. Many researchers have attempted to modulate the biological function of cells by using biomaterials designed with a defined 3D structure and cell-instructive signals enriched with extracellular matrix (ECM)-like components [4–6]. Most ECM molecules have diverse interwoven fibrous structures in the nanoscale range that support cell adhesion and bioactivity, and thus, fabricating scaffolds with an architecture that mimics that of ECM molecules has been an active area of research in tissue engineering [7].

To date, phase separation, self-assembly, and electrospinning have been used to make scaffolds with a nanofibrous architecture [8, 9]. Among them, the electrospinning technique has attracted considerable attention because it offers high porosity and an adjustable pore size distribution in nanofibrous scaffolds. The large surface area and porous structure of electrospun nanofibers allow them to enhance cell functionality after the incorporation of multiple factors [10]. The materials used to fabricate nanofibrous scaffolds are important [11]; synthetic, natural, and composite polymers have been widely used to make electrospun nanofibers [12, 13]. Considerable technical advances in the electrospinning process have enabled the design and synthesis of new polymeric materials with desirable properties, such as the structural variation of nanofibers and the ability to modify their hydrophilicity, conductivity, and antibacterial activity. Many studies have considered the use of nanofiber scaffolds to engineer bone, vascular, neural, and cartilage tissue [14–17].

**Fig. 1:**
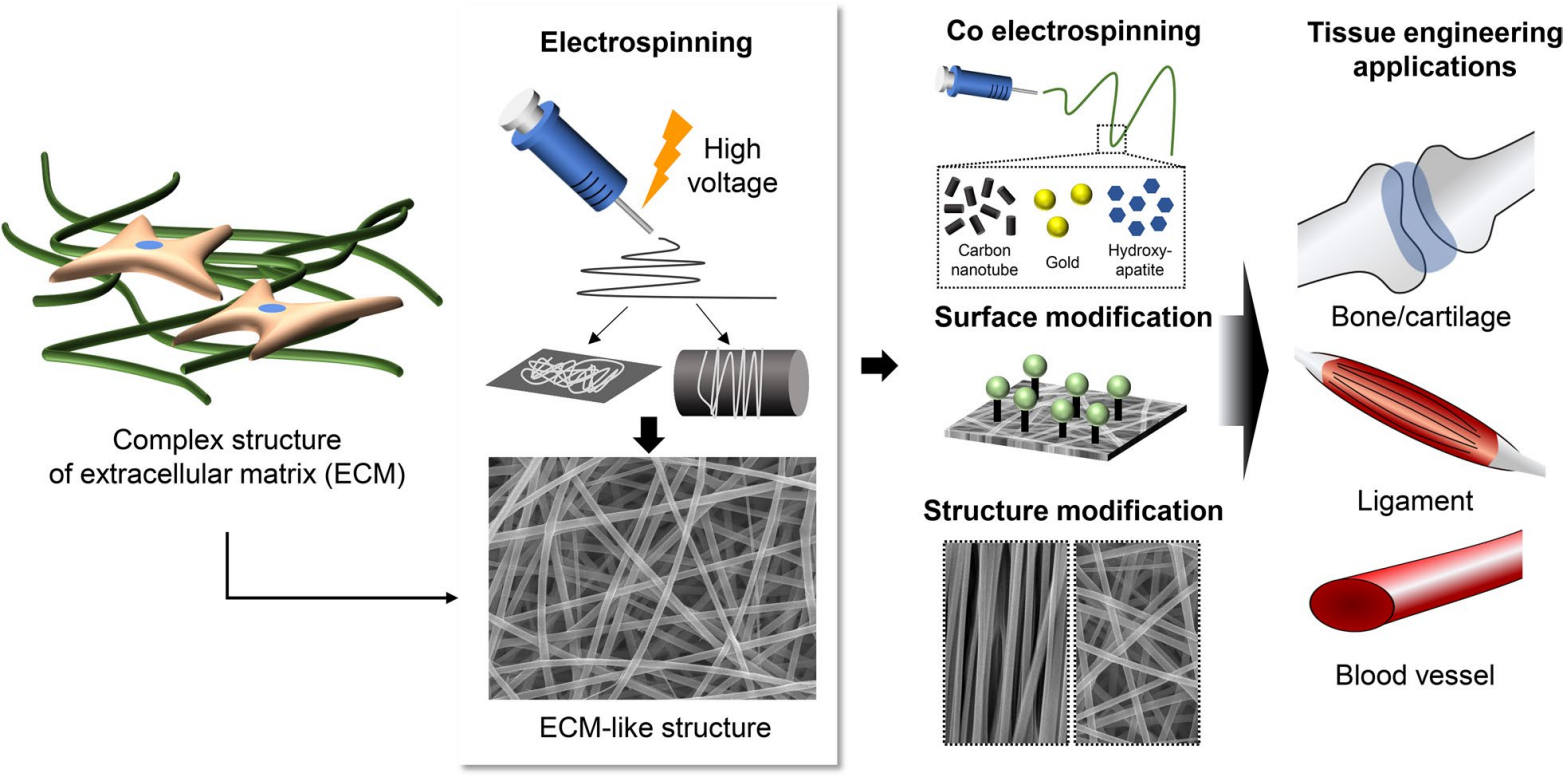
Application of electrospun nanofibers mimicking extracellular matrix (ECM) in tissue engineering

Therefore, in this review article, we have highlighted the great potential of electrospinning for the fabrication of nanofibers to be used as scaffolds in tissue engineering applications. First, we present a brief overview of the different methods proposed for the fabrication of nanofibrous scaffolds, focusing on the electrospinning approach. We then introduce the various types of natural, synthetic, and composite polymers, used in the fabrication of nanofiber scaffolds, highlighting the advantages and drawbacks of each material condition. In addition, we thoroughly discuss different strategies for surface modifications that promote the functionality of nanofiber scaffolds. We also summarize current applications of nanofibrous scaffolds in the regeneration of various types of tissue (Fig. 1).

## General strategies for fabricating nanofibrous scaffolds

ECM is composed of biomolecules such as proteins and polysaccharides that form a complex microenvironment for cells in native tissue [18]. ECM plays several roles, such as supplying mechanical integrity and regulating the signaling processes of cells. For example, sturdy protein macromolecules give the ECM strength and resilience to endure environmental pressure [19]. In addition, ECM proteins are implicated in the dynamic behaviors (migration) and fate decisions (proliferation, apoptosis, and differentiation) of cells through their general mechanical binding with cell receptors such as integrins [20]. Thus, it is critical to design scaffolds that can mimic both the fibrillar configuration and multiple functions of ECM to accelerate cell adhesion, proliferation, differentiation, and tissue genesis [21]. Such an ECM analogue would require topographical characteristics and geometry on the macro-, micro- and nanoscales [22]. Scaffolds composed of nanoscale fibers with a high specific surface area could offer morphological similarities to native ECM. The most well-known techniques for producing nanofibrous structures are self-assembly, phase separation, and electrospinning [8], and their technical details are explained below.

### Polymer self-assembly

**Fig. 2:**
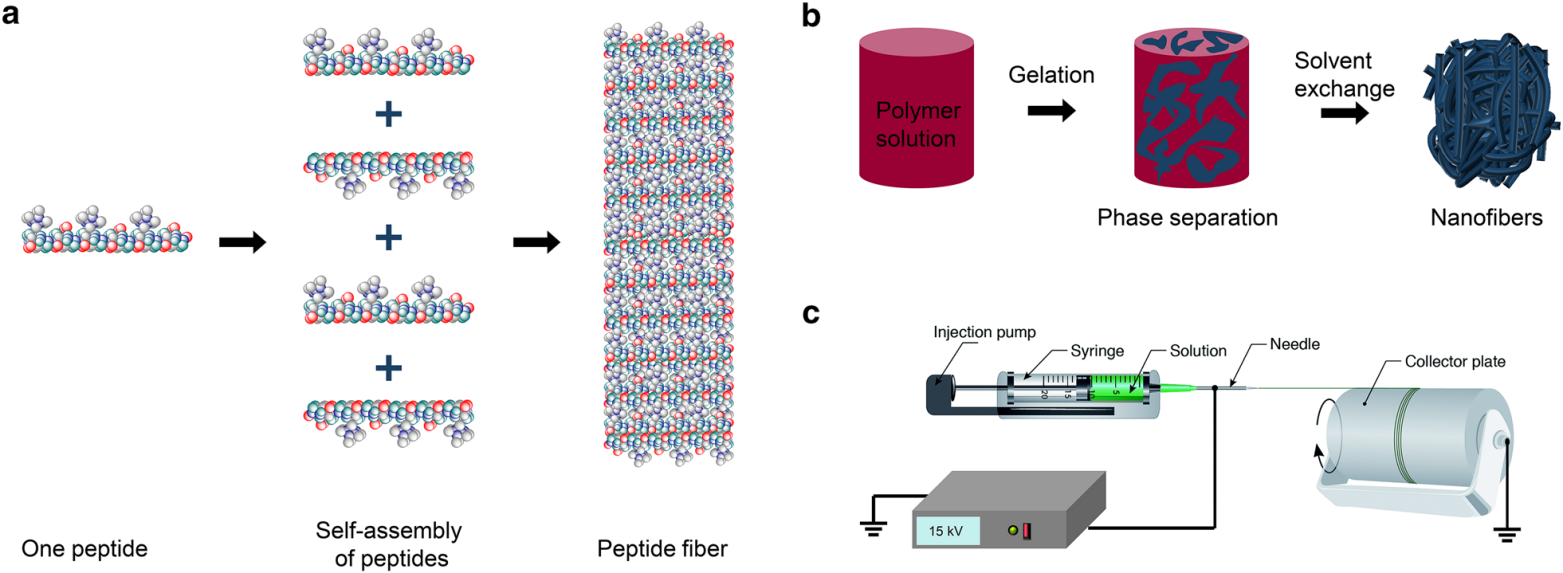
Schematic diagram of nanofiber fabrication techniques. **a** Self-assembly. Reproduced with permission from [24], Nature publication. **b** Phase separation. Reprinted with the permission from [40], Royal Society of Chemistry (RSC). **c** Electrospinning. Reproduced with permission from [50], Royal Society of Chemistry (RSC)

The commonly used self-assembly of polymeric materials involves the intermolecular association of peptides that are immediately assembled into organized, well-defined, stable structures using non-covalent forces such as van der Waals, electrostatic, hydrogen bonding, and π–π stacking interactions. These bonds are generally weak, but when they are combined into a single unit during the assembly process, they control the structural conformation and stability of the assembly and strongly affect the interaction between the supramolecular construction and other molecules, cells, and tissues [23]. The design of peptides with chemical complementarity and structural compatibility is a pivotal parameter for self-assembly, as shown in Fig. 2 [24]. The basic principles for designing novel self-assembling peptides can be optimized by tuning of the amino-acid sequence of the first self-assembling peptide [25]. The presence of electrolytes in the solution can be a driving force for peptide self-assembly. Self-assembling peptides with a slight net positive or negative charge can lead to minor electrostatic repulsion of the peptide monomers. Accordingly, such peptides remain dissolved in water at moderately high concentrations for extended periods of time. Although the peptide self-assembly phenomenon is not fully understood, some studies reported that it occurs based on the lessening of the electrostatic repulsion of the peptide monomers with similar charges induced by electrolyte ions [26]. This phenomenon allows peptides to be placed closer to other peptides, which strengthens hydrophobic interactions and produces stable nanofibers.

Further organization of nanofibers into 3D constructions is accompanied by hydrogel formation and produces pore diameters from 5 to 200 nm [27]. Several parameters involving the sequence of amino acids, peptide solution concentration, hydrophobicity/hydrophilicity, amino-acid charge, concentration and type of electrolyte ions, pH of the solution, and temperature, are known to govern the mechanical properties of a peptide hydrogel [28, 29]. Self-assembling peptide hydrogels are an ideal platform for engineering tissues such as bone [30], nerve [31], cartilage [32], liver [33], cardiac tissue [34], and angiogenesis [35] because they are easy to use, nonimmunogenic, and nontoxic (they do not require harmful chemicals such as a toxic crosslinker). These hydrogels are biodegradable, and their degradation products can be metabolized by cells. Compared with scaffolds produced by the electrospinning method, the nanofibrous structures formed by the polymeric self-assembly process are much thinner. Despite their advantages, self-assembled nanofibers have some limitations: complicated processing, low productivity, and relatively high cost [36]. Moreover, the poor mechanical properties of self-assembled nanofibers (compared with those prepared using other techniques) and the lower kinetic rate of formation with respect to current shear-thinning injectable hydrogels could lead to the in vivo diffusion of self-assembled systems before gelation [37, 38].

### Phase separation

Another technique used to form a porous nanoscale architecture for 3D tissue engineering polymer scaffolds is phase separation (Fig. 2b). This method requires three main steps: dissolution, gelation, and extraction of the solvent from the gel by water, followed by freezing-lyophilization under a vacuum [39]. First, a polymer is dissolved in a solvent at a high temperature, and then a liquid–liquid or solid–liquid phase separation of the polymer is induced through cooling or non-solvent exchange. At that point, the polymer is no longer thermodynamically soluble, so polymer-rich phases are formed within the solvent. Following extraction of the solvent, the scaffold is frozen to maintain its structure. Eventually, highly porous fibrous polymer scaffolds with microscale spherical pores are obtained by lyophilization [40]. Zhang et al. applied a liquid–liquid phase separation technique to fabricate 3D interconnected fibrous networks of poly-l-lactide (PLLA) to mimic the nanofibrous structure of ECM. The fabricated fibers had diameters of 50–500 nm, analogous to that of collagen matrix [41, 42].

Several parameters, such as the type of polymer and solvent, polymer concentration, gelation temperature, gelation duration, and thermal treatment, have been reported to influence the nanofiber morphology [43]. One important parameter is the selection of a suitable solvent. Liu et al. [44] used ethanol/water and methanol/water solvents for the formation of a nanofibrous structure of gelatin, in which ethanol and methanol served as non-solvents to manipulate the interactions between the gelatin and solvent molecules and subsequently control the phase separation conditions. Notably, solvent mixture systems, such as acetone/water and dioxane/water, did not provide nanofibrous matrices due to poor interactions between gelatin and solvent molecules. The polymer concentration can affect the structure of nanofibrous matrices, including the diameter of fibers, scaffold porosity, and fiber length between conjunctions. Nanofibrous PLLA scaffolds with a PLLA concentration of 7% had a porosity of 38%, and decreasing the PLLA concentration to 5% increased the porosity to 89% [45]. Pore morphology and the porosity of the scaffolds made from different types of polymers can be considerably tuned. For example, PLLA and poly (lactide-co-glycolide) (PLGA) displayed distinct porosities and pore sizes in the same experimental conditions. The PLGA scaffolds had smaller pore sizes than the PLLA-based ones due to the rearrangement of polymers chains during the phase separation process [46].

By using specific molds, the phase separation process enabled the fabrication of 3D scaffolds with designated dimensions. Using computer-aided design for the molds can enable the creation of complex architectures, such as a human ear or jawbone [40]. Scaffolds formed using phase separation resemble more conventional foams, with large pore sizes that can improve cell infiltration [40]. Zhao et al. [47] used a dual thermally induced phase separation method to create nanofibrous PLLA scaffolds decorated with a chitosan nanofiber network inside macro-pores (300–450 µm). The bone marrow stromal cells cultured on those scaffolds for 14 days demonstrated greater viability than the cells on traditional PLLA scaffolds. Although the phase separation approach offers advantages such as simplicity, low cost, and ease of fabrication, it also has drawbacks, including long processing time, laboratory scale production of nanofibers, instability of the nanofiber structure, difficulty in controlling the porosity, and being limited to polymers amenable to phase separation processes [48].

### Electrospinning

Electrospinning, which uses electrostatic forces to generate synthetic fibers, has gained intense interest among tissue engineering researchers ever since it was reported that the structural properties of materials play a crucial role in controlling cell behavior [49]. A typical electrospinning setup generates an electric field between a counter electrode and a positively charged spinneret filled with a polymer solution, as shown in Fig. 2c [50]. A polymer jet is formed at the metal needle tip because the electrostatic charge is larger than the surface tension of the polymer solution. Subsequently, the polymer jet moves through the charged spinneret to the counter electrode, allowing the solvent to evaporate and forming continuous polymer fibers with diameters of tens of nanometers to a few micrometers (depending on the polymer solution and electrospinning parameters) that gather on the collector [51]. The electrospinning variables that affect fiber formation and structure include solution parameters (polymer concentration, solvent volatility, and solution conductivity) and processing parameters (flow rate, needle to collector distance, and applied voltage) [52]. Among them, the polymer concentration plays a significant role in the formation of stable fibers, noted as spinnability, which largely depends on the entanglement of polymer chains in the solution. The viscosity and surface tension of the solution are also affected by the polymer concentration. For example, dilute solution produces mechanically weak fibers or polymer droplets, and a solution that is concentrated beyond a critical value impedes the formation of fibers at all [53]. It has been frequently reported that the fiber diameter increases with the polymer concentration within the optimal range. Shao et al. [54] fabricated randomly oriented electrospun poly (vinylidene fluoride) (PVDF) nanofiber mats and reported that increasing the PVDF concentration from 16 to 26% increased the diameter of the resulting fibers from 0.2 to 0.8 nm.

Multiple experimental arrangements of the electrospinning process, in addition to solution and processing parameters, have been used to modify the primary properties of the fibers, such as nozzle configuration (coaxial nozzles) or solution vs. melt spinning. Coaxial electrospinning uses two aligned needles that can concurrently spin two different polymer solutions. Both metal syringes are operated under a similar voltage, which deforms the compound droplet on the tip. Core–shell nanofibers, in which a large fiber encapsulates a smaller fiber, were generated when a jet was formed on the deformed droplet tip. By applying appropriate solution concentrations, core–shell nanofibers have been produced with high precision [55]. If the shell fluid is at too high at concentration, creating a proper morphology of the nanofiber through coaxial electrospinning becomes difficult. Thus, forming coaxial composite nanofibers with excellent morphology requires that the concentration of the solution be as low as possible [56]. Continuous core and hollow fiber structures can also be generated by coaxial electrospinning. Continuous core fibers are produced by electrospinning a coaxial jet of a polymer that is hydrophilic on the outside and hydrophobic on the inside [57]. A hollow fiber structure can be produced by precipitating the core polymer on the wall of an already formed shell upon evaporation of the solvent [58]. However, despite the desirable mechanical properties and complex structures of electrospun nanofibers, the co-electrospinning technique offers poor control [59–61].

Melt electrospinning is an alternative approach that side-steps the toxicity and accumulation of solvent that can be issues with coaxial electrospinning [62]. In this method, a high electric field is generated between the metal-based collecting rotor and the polymeric melt within the heated extruder. In other words, the polymer is first melted, and then the nanofibers form during the phase change induced by cooling. Consistent with the coaxial electrospinning technique, the driving force responsible for fiber generation is the attenuation of a spin line exposed to electrostatic forces. During transit, the jet diameter decreases continually because of the electrostatic forces acting on it until the viscosity overcomes the electrostatic forces as the jet solidifies as it cools [63]. It has been reported that fiber diameter can be adjusted by controlling the processing parameters, such as polymeric viscosity, electric field strength, and flow rate. Environmental safety and higher productivity are advantages of this method; however, the elevated temperatures required for melt electrospinning might not be compatible with the incorporation of bioactive molecules or drugs within the nanofibers [64].

## Materials for electrospun nanofiber fabrication

Many synthetic and natural polymers have been used in the design of nanofibrous scaffolds with different structural properties. Synthetic polymers generally provide high flexibility in synthesis, processing, and modification and are more cost-effective than natural ones. Importantly, their mechanical properties can be effectively and preferentially tuned. However, synthetic polymers lack bioactivity and thus require more modifications than natural ones. On the other hand, natural polymers are inherently bioactive, presenting cell-interactive domains on their backbones, and scaffolds prepared using them offer better adhesion, proliferation, and differentiation of cells than is available with synthetic polymers. Of particular note, the products of degradation from natural polymers are chemically benign and elicit relatively low immune responses [65]. To exploit the advantages of both synthetic and natural polymers, researchers have fabricated hybrid scaffolds with the physical properties and high bioactivity favorable for tissue regeneration [66].

### Natural polymers

Natural polymers, such as collagen, gelatin, chitosan, fibrinogen, hyaluronic acid, and silk, have often been used as scaffolds because of their biocompatibility and bio-functionality [67–69]. To enhance the strength of electrospun natural polymers and retain their fibrous form, crosslinking is commonly used [70]. Collagen nanofibers can be crosslinked by different methods during or after electrospinning, including stabilization with glutaraldehyde vapors, formaldehyde, or epoxy compounds and exposure to ultraviolet light [71]. Zhou et al. [69] prepared electrospun tilapia collagen nanofibers and demonstrated that the tensile strength of the collagen nanofibers crosslinked by glutaraldehyde vapor was 6.72 ± 0.44 MPa, making them suitable for use as artificial human skin. Nanofibers of gelatin, a denatured form of collagen, have been also stabilized by chemical crosslinking (using glutaraldehyde, carbodiimides, and genipin) or physical blending with other polymers. For example, genipin-crosslinked electrospun gelatin scaffolds exhibited an average diameter of 570 ± 140 nm and retained a more fibrous structure than non-crosslinked ones, showing restricted fused regions where the fibers overlapped [72]. Chitosan is a polysaccharide prepared by the deacetylation of chitin, and acidic solutions, such as diluted hydrochloric acid, acetic acid, formic acid, and trifluoroacetic acid, have been used in electrospinning it. Its antibacterial and hemostatic properties, in addition to its low immunogenicity and biocompatibility, made the resulting scaffolds suitable for wound healing applications [73]. The electrospinning of fibrinogen has also been reported, producing electrospun fibrinogen fibers with an average diameter of 95 nm that were more extensible and elastic (ɛ_elastic_ = 16%) than other natural electrospun fibers and had the same stiffness such as collagen (ɛ_elastic_ < 2%) and a strain of 74% under tensile stress of 2.1 GPa, which might be stable enough for tissue applications [74]. Similar to other natural polymers, crosslinking fibrinogen scaffolds improved their mechanical strength and decreased the rate of degradation. For example, electrospun fibrinogen crosslinked with 1-ethyl-3-(3-dimethylaminopropyl)carbodiimide and genipin retained its mechanical integrity for 14 days [75].

### Synthetic polymers

Numerous synthetic polymers have been applied to the construction of electrospun scaffolds. The main advantages of synthetic polymers are their spinnability, excellent mechanical integrity, and cost-effectiveness. Polyesters such as poly (e-caprolactone) (PCL), polylactic acid (PLA), polyglycolic acid (PGA), and polyglycerol sebacate and polyethers such as polyethylene oxide (PEO), polyurethanes, and functionalized polyolefins [polyvinyl alcohol (PVA)] have been electrospun for tissue engineering applications [76]. PCL is a promising and frequently used biodegradable synthetic material with high tensile strength due to its distinct rheological and viscoelastic properties, making it an appropriate candidate for applications in which mechanical strength is important. PCL fibers with an average diameter of 1833 ± 369 nm were fabricated by Gomes et al. [77]. The PCL scaffolds showed elasticity of 6.7 ± 0.4 MPa and ductility of 587 ± 162%, demonstrating their great potential for tissue engineering applications. In another study, electrospun nanofibrous esophageal tissue scaffolds made of PCL had a tensile strength of 6.8 ± 0.81 MPa, which is higher than that of a cadaveric esophagus (1.289 ± 0.517 MPa), indicating their suitability for esophageal regeneration [78].

PLA, PGA, and the copolymer PLGA have been used to fabricate electrospun nanofibers for applications that demand faster degradation rates and natural degradation products. PLA can hydrolytically degrade into lactic acid, making it appropriate for medical applications. A high surface area, biomimicry of native ECM structure, and proper mechanical properties are other advantages of PLA nanofibrous scaffolds [79]. PGA, which degrades into glycolic acid at a faster rate than PLA, is a thermoplastic polymer with good resorbability and high applicability as a support matrix in rebuilding neuronal tissue or producing structures for controlled drug release [80]. Moreover, the high specific surface area and porosity of nanofibrous PGA scaffolds can facilitate the diffusion of degradation products and consequently increase the rate of degradation [81]. PEO, a hydrophilic polymer, can be used for applications that require soft tissue-like mechanics, but due to its high susceptibility to dissolution upon hydration, crosslinking after electrospinning is needed [82]. Therefore, developing synthetic polymers mimicking such a biocomplexity behavior is of paramount importance in order to raise the possibility of utilizing synthetic scaffolds in this area [83].

### Composite nanofibers prepared with multiple materials

A blend of polymers can be applied to overcome the limitations of mono-component systems. This strategy can produce new tissue engineering scaffolds that possess optimal mechanical and biological features. In general, synthetic polymers provide high mechanical strength, and natural polymers on the surface or inside the scaffolds enable the cell-recognition signals that are vital for cell behavior and improvement [84]. For example, Zhang et al. [85] investigated the properties of PCL/collagen composite fibrous scaffolds (PCFSs) and found that the proliferation of L929 fibroblasts on PCFSs was high, and their tensile strength was around 2.02 MPa, suggesting that they had sufficient mechanical integrity through the synergistic effects of PCL and collagen. Gelatin blended with natural or synthetic polymers exhibited physicochemical, biomechanical, and biocompatibility properties attractive for scaffolds [84]. In addition, biopolymer blends of PVA and chitosan have been fabricated as nanofibrous scaffolds by the free-surface electrospinning technique and showed potential for numerous tissue engineering applications. Agrawal et al. [86] reported that the optimum chitosan/PVA ratio (35:65) produced a high tensile strength (6.15 MPa) because of its viscosity. Those results suggested that the scaffold was free from any adverse conditions, promoting cell proliferation and growth on the scaffold through the biocompatibility of the chitosan. Similarly, an electrospun PCL/chitosan scaffold was shown to be an appropriate matrix for an engineered scaffold with good mechanical properties (elastic modulus: 7.8 ± 0.5 MPa). The resulting fibers displayed 89.9% of liver mouse cell (Hepa 1–6) viability, indicating the cell nontoxicity and compatibility of the PCL/chitosan nanofiber scaffold [87].

## Preparation of functional nanofibers

Despite the advantages of electrospun nanofiber scaffolds, their chemical, biological, and mechanical properties often require further modification on the surface and structure to promote their functionality. It is well-known that material features significantly affect the interaction between cells and a scaffold. For example, a specific material might be good choice for scaffold formation but not be suitable for cell growth because of its surface or bulk properties. In this regard, co-electrospinning with additives such as biological materials or conductive elements, post-treatment of surface characteristics, and controlling the dimensions and arrangement of the fibrous structure of electrospun fibers have been widely investigated, as explained below.

### Co-electrospinning with additives

Incorporating biological materials within a basic polymeric substance and then electrospinning the blend (co-electrospinning) is a well-known method for improving the specific biological functionality of a polymer. The resulting mixture efficiently improves the attachment, proliferation, viability, and differentiation of cells [88]. For example, Gopinathan et al. [89] functionalized PCL nanofibrous scaffolds by incorporating galactose biomolecules, demonstrating that the DNA content of the cultured meniscal cells had increased approximately 1.6-fold in a galactose-base scaffold on day 24, with improved secretion of collagen and glycosaminoglycan. Scaffold conductivity has been improved by including conductive and biocompatible additives such as carbon nanotubes (CNTs) and gold [90, 91]. For example, CNTs have been used for electrical and mechanical strengthening, particularly for neural, muscle, and bone tissue engineering [92]. Electrospun carboxyl multi-walled carbon nanotube-grafted polyhydroxy butyrate (CMWCNT-g-PHB) composite nanofiber scaffolds were fabricated. An increase in the CMWCNT content in the composite nanofiber scaffolds increased the alkaline phosphatase (ALP) enzyme-related activity of MG-63 human osteoblast cells by 260% [93]. In addition, Motamedi et al. [94] reported that adding gold nanoparticles (AuNPs) to PVDF as a composite scaffold made cells more elongated and spread‐out after culturing for 24 h by mimicking the morphology inside nerve tissue, potentially through the improved piezoelectricity of the prepared scaffolds by means of the high AuNP-mediated conductivity.

**Fig. 3:**
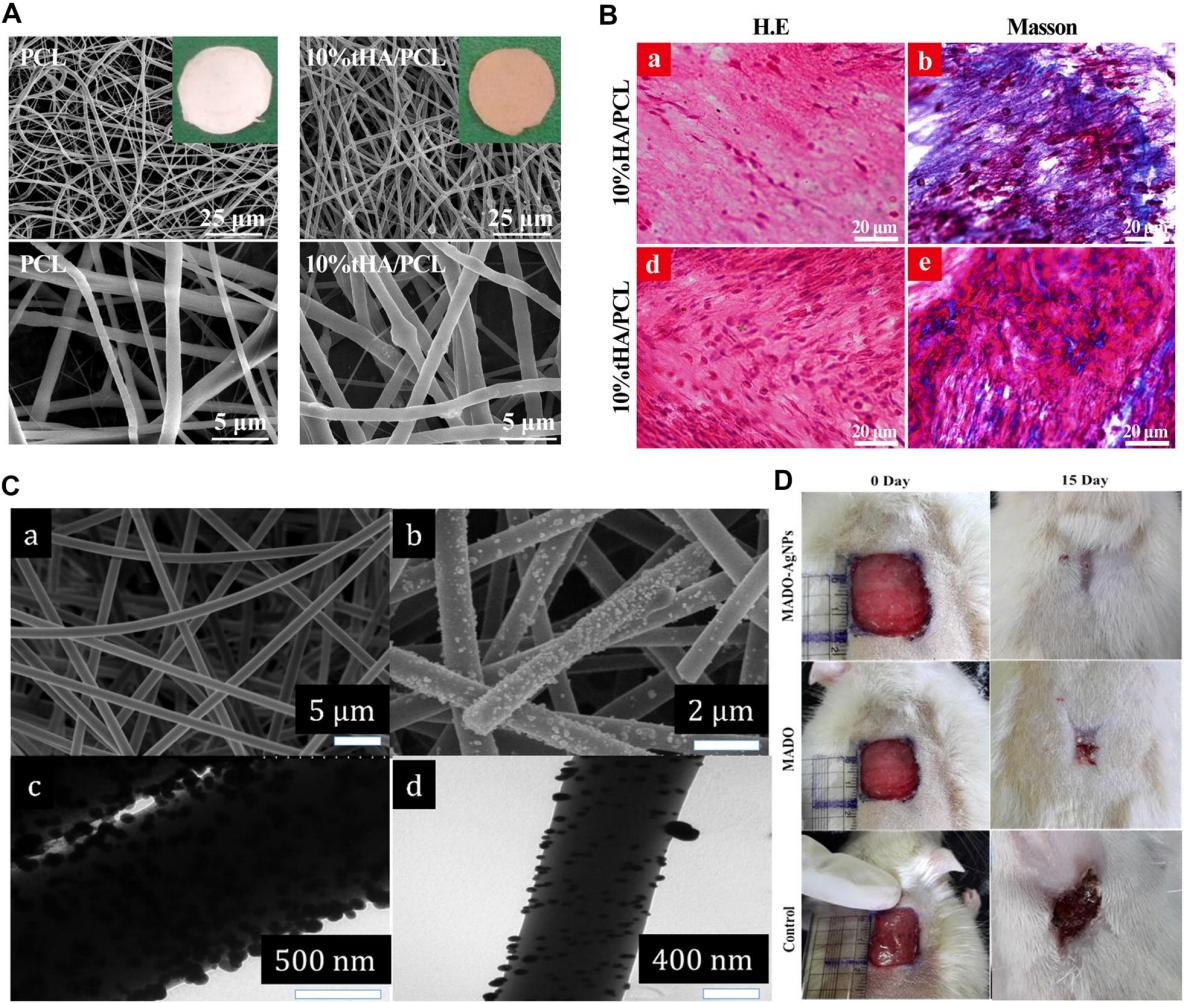
**A** SEM images of PCL and 10% tHA/PCL nanofibers. **B** H&E and Masson staining of hMSCs-10% HA/PCL (top) and hMSCs-10% tHA/PCL (bottom). Reproduced with permission from [96], American Chemical Society (ACS). **C** FESEM images of electrospun (top, left) MADO nanofibers and (top, right) MADO/AgNPs-coated nanofibers. TEM images of MADO/AgNPs-coated nanofibers after 24 h (bottom, left) and 12 h (bottom, right) of incubation, respectively. **D** Wound healing at 0 and 15 days after implantation of MADO/AgNPs-coated nanofibers (top), MADO (middle), and pristine sample (bottom). Reprinted with permission from [101], American Chemical Society (ACS)

Electrospinning polymers while incorporating bioactive inorganic nanoparticles such as hydroxyapatite (HA) and calcium phosphate (CaP) has also been used to generate scaffolds with enhanced mechanical properties and osteoconductive features because of the physicochemical similarity in structure and composition between HA and CaP and natural nanocrystals in bone tissue [95]. For example, novel nano-HA plates were embedded in PCL nanofiber via co-electrospinning (Fig. 3A). The enhanced elastic modulus and osteoconductivity of this composite scaffold induced the osteogenic differentiation of human mesenchymal stem cells (hMSCs) in in vitro and promoted bone formation in in vivo studies (Fig. 3B) [96]. In another study, HA/poly (butylene adipate-co-terephthalate) nanofibers were fabricated, and the presence of HA not only enhanced the elastic modulus by 12.7%, the stress at break by 25.7%, and the elongation at break by 52%, but also ensured the differentiation of human adipose stem cells (hADSCs) [97]. Ko et al. [98] fabricated electrospun silk fibroin nanofibrous scaffolds functionalized with HA in both inner and outer parts, which considerably improved the mechanical properties (Young’s Modulus, almost 5 kPa). These HA-functionalized scaffolds transplanted in the critical-sized calvarial bone defect model effectively accelerated the formation of mineralized bone. Also, the deposition of CaP on hydroxyethyl cellulose/PVA nanofibers improved their elastic modulus from 355 ± 10 to 412 ± 12 MPa and the osteoconductivity of the scaffolds [99]. Incorporating inorganic nanoparticles such as silver salts or magnetic particles is a well-known way to improve the antibacterial properties of engineered scaffolds [100]. Ghavaminejad et al. [101] fabricated poly (dopamine methacrylamide-co-methyl methacrylate) (MADO) nanofibers functionalized by silver nanoparticles (AgNPs) (Fig. 3C). The AgNPs diffused freely into the test media, interacted with the sulfur-containing intracellular proteins in bacteria, and consequently demonstrated antibacterial properties, which can be useful in wound healing, as shown in Fig. 3D.

### Surface modification of electrospun nanofibers

Due to the relatively inert chemical properties of synthetic polymers, tuning their surface properties (wettability, hydrophilicity, and cell adhesion) remains challenging. Modifying the nanofiber surface after electrospinning is critically important because it makes them more compatible with tissue engineering [102]. The surface properties of nanofibers can be modified chemically and physically with bioactive molecules and cell-recognizable ligands. Plasma treatment has generally been used for the surface modification of electrospun nanofibers prepared from synthetic polymers [103]. The introduction of higher-oxidation-state carbon species that contain alcohol, ether, and carbonyl groups can functionalize the surface of electrospun nanofibers. The resulting functional groups alter the surface characteristics, including wettability, polarity, and protein adsorption, thereby improving cellular behavior through enhanced polar interactions induced by the polar functional groups [104]. For instance, amine and carboxyl/anhydride plasma coatings on the surface of PCL nanofibers considerably improved the cell adhesion and viability of mouse myoblast C2C12 cells [105]. Oxygen plasma treatment of PLLA nanofibers was also found to significantly affect the initial adhesion of porcine mesenchymal stem cells, as assessed by cell capture efficiency. The cell capture percentage of plasma-treated PLLA during a 10-min capture time was about 3 times higher than that of bare PLLA (15% vs. 5%). This was mainly ascribed to the hydrophilic characteristics conferred by the oxygen-based polar groups [106].

**Fig. 4:**
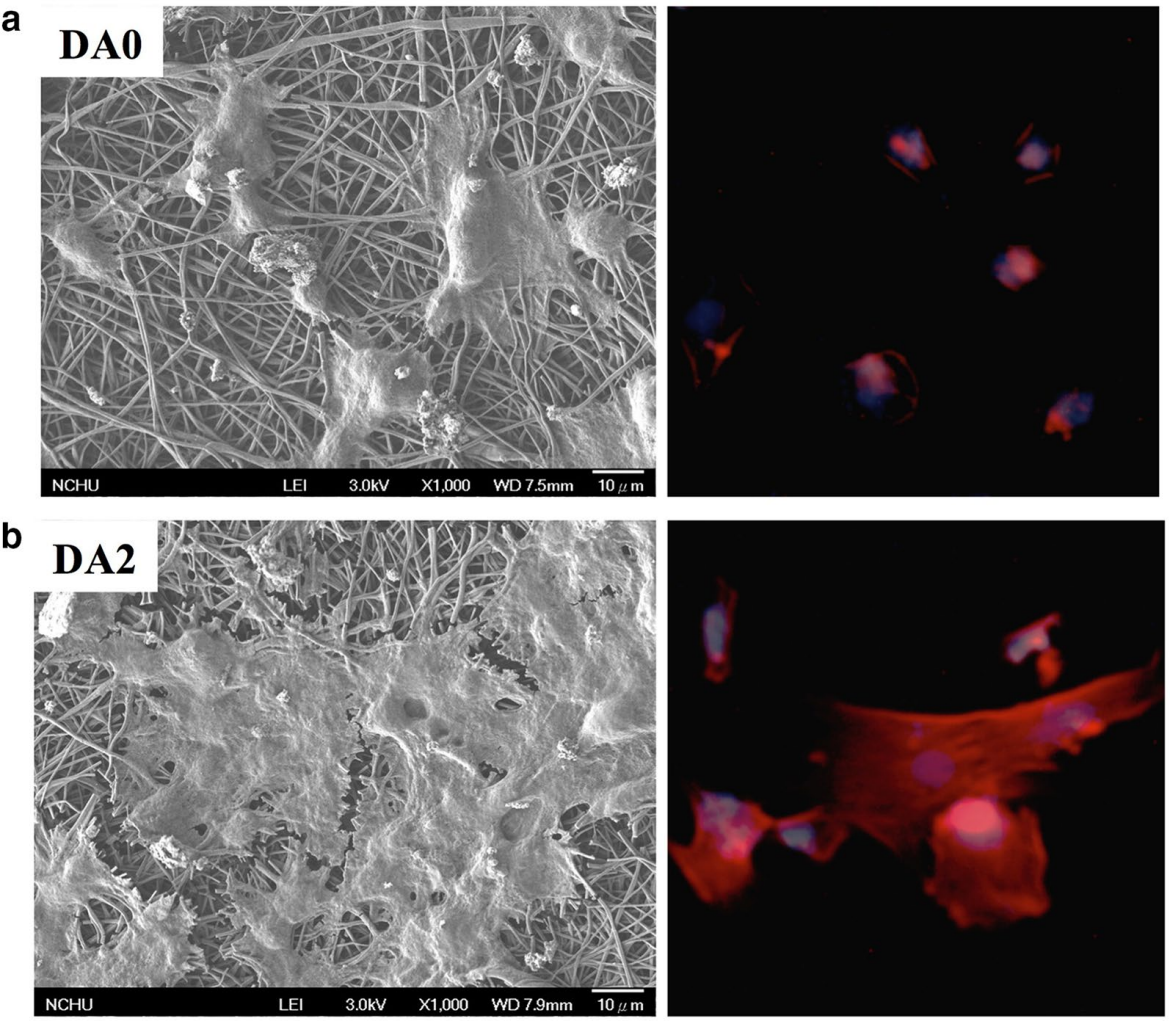
**a** SEM and immunofluorescence images of hADSCs attached on PLA and **b** PDA/PLA nanofibers. Reproduced with permission from [109], Elsevier

A poly(dopamine) (PDA) “mussel-inspired” coating is another method for modifying the surfaces of electrospun fibers [107]. It has been discovered that dopamine has alkylamine and catechol groups that could undergo self-polymerization in alkaline conditions and form PDA. The formation of PDA layers on polymeric materials such as PLLA and PCL is expected through hydrogen bonding, π–π stacking, and van der Waals interactions, and that PDA layer could allow covalent reactions with different functional groups, potentially by reacting with both thiols and amine functional groups via the Schiff base reaction and Michael addition [108]. Therefore, PDA chemistry could easily modify the surface properties of electrospun nanofibers, including their chemical functionality, hydrophilicity, roughness, and mechanical properties [108]. PDA coating on PLA electrospun nanofibers facilitated the adhesion of hADSCs with respect to non-treated PLA (Fig. 4a, b) [109]. In another study, electrospun PLA/cellulose nanofibril (CNF) composite nanofibers were treated with a dopamine solution. The hydrogen bonding between the CNF and PDA molecular chains facilitated the deposition rate of the PDA coating layer on the surface of the composite nanofiber. The PDA-coated PLA/CNF scaffolds showed better performance in terms of the proliferation and growth of hMSCs than the pristine PLA/CNF one, along with improved mechanical properties and hydrophilicity [110]. Lee et al. [111] reported that a PDA coating on PCL nanofibers scaffolds promoted the HA crystallization and deposition on the scaffold, which improved both mechanical properties of the PCL scaffolds and bone regenerations.

The layer by layer (LBL) technique has also been used to coat the surfaces of electrospun nanofibers with natural materials and biomolecules. This method uses the alternative adsorption of oppositely charged polymers to create a self-assembled multilayer coating with adjustable thickness and properties, including charge, porosity, and chemical functionality, while avoiding the denaturation and partial activity loss of bioactive molecules. Deposition on charged substrates is caused by electrostatic forces, hydrophobic interactions, hydrogen bonding, and covalent binding [112]. The LBL method was used to coat polyacrylonitrile and PLGA with triple-helical anionic and cationic collagen. L929 mouse lung fibroblast attachment and spreading were enhanced because the collagen retained its native triple helical structure [113]. In a different study, the surfaces of PLLA ultrafine fibers were activated by treating them with poly (ethylene imine) (PEI), adsorbed laminin (LN), and chitosan using LBL assembly. A positive charge was generated by amine etching on the surface of PLLA electrospun ultrafine fibers via the reaction of the amine group of PEI and the ester group of PLLA, followed by the alternate adsorption of negatively charged LN and positively charged chitosan. The modified PLLA ultrafine fibers showed greater neuronal growth and neurite outgrowth of dorsal root ganglia neurons than unmodified PLLA nanofibers [114].

### Structure modification of electrospun nanofibers

Developing scaffolds that mimic the architecture of natural human tissues at the nanoscale is a major challenge, but a well-defined structure is essential for the scaffold to properly imitate native ECM in guiding cell growth or tissue regeneration. The electrospinning technique has attracted important attention because of its potential to fabricate fibers similar to the fibrous structures of native ECM. Moreover, when the structural and biological features of the native tissues are understood, nanofibers can be electrospun in various patterns for the best reconstruction based on the structure of the tissue to be engineered [115]. Aligned electrospun nanofibers can easily be produced by orienting the fibers in one direction through injection onto a collector at a high speed (more than 1500 rpm). The shape of nanofibers is important in tissue engineering applications because some tissues, such as tendons, heart tissue, and blood vessels, exhibit anisotropic arrangements with highly ordered structures. As expected, electrospun nanofibrous scaffolds with suitable alignments have been found very effective in shaping cell morphology, guiding cell migration, and controlling various levels of cell behavior [116]. For example, cardiomyocytes cultured on aligned electrospun PCL nanofibers showed cellular alignment and elongation within the scaffold, which enhanced their maturation [117]. In another study, Cho et al. [118] demonstrated that PCL aligned nanofibers provided structural guidance for the growth of the induced neuronal cells and also served as pseudo-axonal scaffolds to promote the formation of myelin-like segments from induced pluripotent stem cell derived oligodendrocyte.

A nanofibrous scaffold that contains both aligned and random sections could mimic the structural arrangement of collagen fibers at the tendon-to-bone insertion area, with the aligned portion analogous to collagen fibers in a normal tendon and the random portion resembling collagen fibers in bone. Tendon fibroblasts cultured on the aligned portion of such a nanofiber scaffold possessed highly organized morphology, whereas those on the random portion showed a haphazard morphology [119]. Park et al. [120] used electrospinning to develop a novel hybrid scaffolding system that combined aligned fibers (AFs) and random fibers (RFs). The AF layer in the upper part of the scaffold offered uniaxial topographic guidance and enabled the preferential alignment and differentiation of cultured C2C12 myoblasts, whereas the RF layer (lower part) provided support and adequate mechanical properties. Cai et al. [121] prepared dual-layer aligned-to-random poly (l-lactic acid-co-e-caprolactone) (PLCL) scaffolds using an electrospinning process. The failure load and stiffness of the aligned-to-random scaffolds 12 weeks after surgery were about 83 N and 21.5 N/mm, considerably higher than those reported for a random scaffold (66.2 N and 15.6 N/mm) and the control group (50.6 N and 10 N/mm).

## Applications of electrospun nanofibers in tissue engineering

### Vascular tissue engineering

Many approaches have been used for the in vitro construction of fully functional vascular tissue with the potential to interact with cells at the nanoscale, leading to blood vessel formation. For example, keratin was electrospun with PCL to fabricate nanofibrous mats for use as vascular tissue. These materials were more favorable for the attachment of NIH 3T3 cells than pristine PCL, mainly because of the presence of cell adhesion moieties in the keratin, such as RGD (Arg-Gly-Asp) and LDV (Leu-Asp-Val), along with the more hydrophilic properties of the PCL/keratin mats. In addition, the nanofibers demonstrated a lower activated thromboplastin time compared with the platelet-poor plasma control group, with a reduced hemolytic rate for red blood cells [122]. The electrospinning technique offers accurate control over the alignment of fibers to modulate the porosity, pore size distribution, and structure of the scaffolds. In particular, aligned nanofibers that mimic the structural assembly of endothelial cells and smooth muscle cells in the intima and media, respectively, have been examined [123]. Aligned electrospun PE scaffolds blended with elastin and collagen supported the growth of smooth muscle cells with a contractile phenotype and a Young’s moduli of 42.32 ± 8 MPa, which was within the range of native arteries [124]. In addition, acrylamide-terminated RGD was used to alter a poly (ester-urethane) urea (PEUU) electrospun nanofibrous scaffold, which was followed by a covalent immobilizing technique to improve endothelialization. The PEUU-RGD nanofibers mats exhibited a low hemolysis rate (1.21%) and promoted the attachment and retention of endothelial cells with rapid endothelialization [125].

**Fig. 5:**
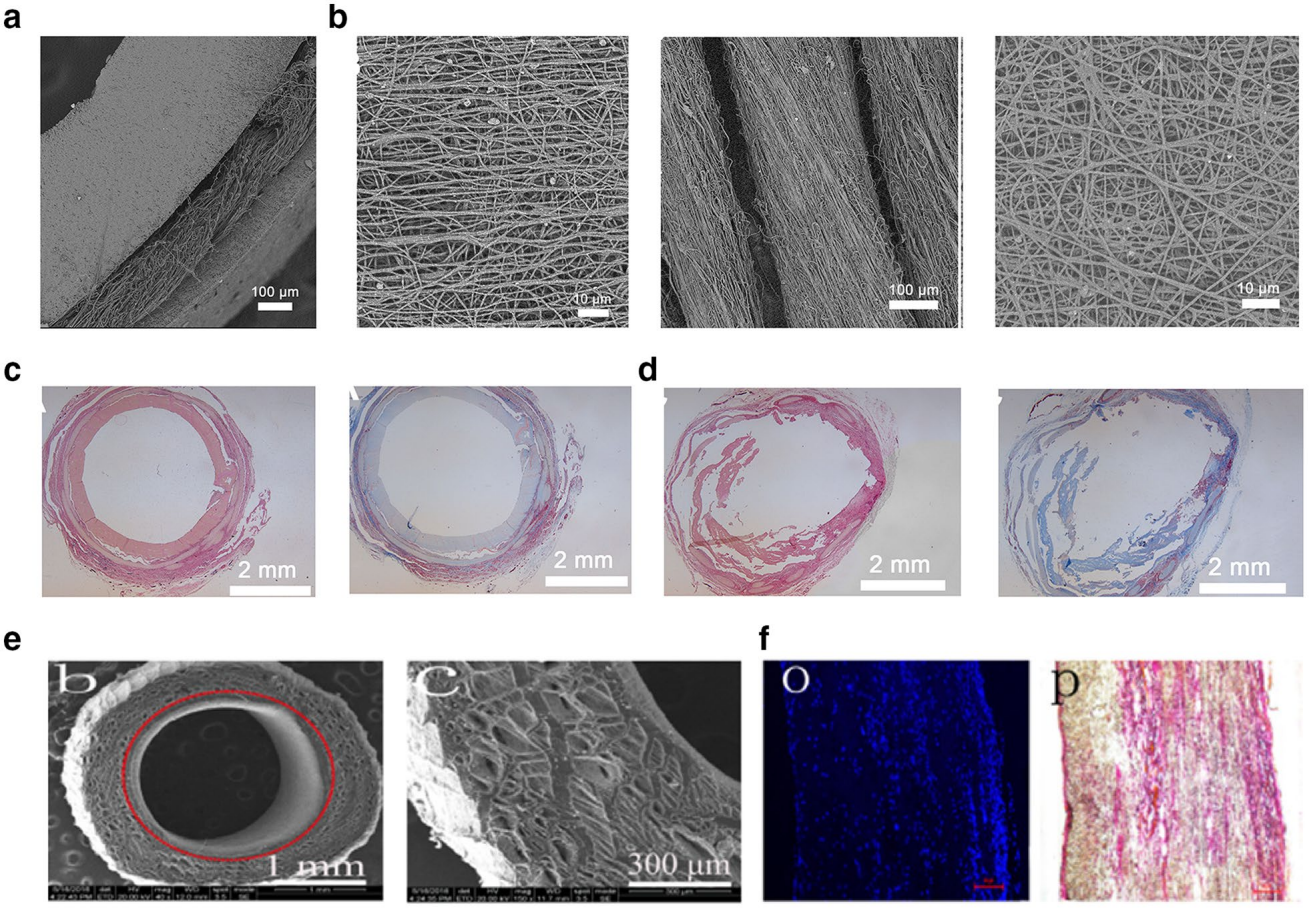
**a** SEM images displaying a cross-section of a tri-layered tubular graft. **b** The aligned structure of PLCL/COL nanofibers in the inner layer (left), the PLGA/SF in the middle layer (middle), and randomly oriented PLCL/COL nanofibers in the external layer (right). **c**, **d** H&E (left) and Masson’s trichrome (right) staining images of transplanted grafts after 2 weeks and 10 weeks, respectively. Reprinted with permission from [126], Elsevier. **e** Cross-sectional SEM image of vascular grafts and relevant high magnification (right). **f** DAPI (right) and H&E staining (left) of bilayer scaffolds implanted in Sprague–Dawley rats after 16 weeks. Reproduced with the permission from [127], Elsevier

A multi-layered vascular scaffold could be an excellent way to generate a tissue-engineered vascular graft because multi-layered scaffolds can easily mimic the structure of natural blood vessels. Wu et al. [126] developed a novel tri-layer tubular scaffold (Fig. 5a) composed of axially aligned PLCL/collagen (COL) fibers, with an in inner layer 336.9 ± 107.27 nm thick to support the tubular structure and prevent blood leakage, oriented PLGA/silk fibroin (SF) yarns in the middle layer (206.17 ± 46.23 µm thick), and random PLCL/COL fibers in the outer layer (361.15 ± 139.91 nm thick) to stabilize the yarns on the inner layer, as shown in Fig. 5b. The results indicated excellent mechanical performance because the PLCL/COL aligned in the parallel direction, single PLGA/SF yarn, and random PLCL/COL fibers had an ultimate stress of 23.03 ± 2, 15.57 ± 1.33, and 15.73 ± 2.55 MPa and largest elongation of 103.75 ± 5.3%, 201.14 ± 17.16% and 99.1 ± 14.57%, respectively. The H&E staining (Fig. 5c) and Masson’s trichrome (Fig. 5d) images of the transplanted grafts after 2 and 10 weeks clearly confirm that the tri-layer vascular graft was able to regenerate the tissue. In another study, Tan et al. [127] produced a bilayer PCL fibrous vascular graft (Fig. 5e) with hemolysis values of 0.67 ± 0.03%, far less than the safe limit (5%). The Young’s modulus of 1.93 ± 0.36 and tensile strength of 4.5 MPa made those scaffolds plausible for vascular tissue engineering because they provided adequate in vivo support for cells to grow into mature vascular tissue, and they provided good endothelialization after 16 weeks, as shown in Fig. 5f. Yu et al. [128] fabricated hybrid vascular grafts from SF and thermoplastic polyurethane (TPU) and using an aligned inner layer and random outer layer. The cyclic circumferential tensile test of the TPU/SF grafts indicated mechanical properties comparable to those of native coronary arteries.

### Neural tissue engineering

Although autografting and synthetic nerve grafts have been clinical options for remedying peripheral nerve injuries, autografting lacks available donor, and synthetic nerve grafts have low proficiency in rebuilding nerve defects [129]. The use of scaffolds from electrospun nanofibers could be an alternative strategy for the treatment of peripheral nerve injury. Electrospun nanofibers provide improved neurite outgrowth, the ability to design nanofiber-based artificial nerve grafts, and mechanical and biochemical cues for differentiating stem cells [130]. A nanofiber scaffold for nerve regeneration should provide adequate surface area for Schwann cells growth and migration with directing the axons’ elongation. Due to axial orientation structure of axon, some researchers have recommended the topographical cues of aligned structure which can provide better contact guidance toward neurite outgrowth. Hu et al. [131] seeded PC12 neural-like cells on electrospun PCL aligned and random nanofiber scaffolds and found that the aligned-PCL scaffold increased the length of the neurites and guided the neurite extension parallel to the fiber axis.

An effective cue to promote neurite and axonal outgrowth and enhance nerve cell growth and differentiation could be electrical stimulation. Electrically conductive CNTs, graphene (Gr), or polyaniline, which has less cytotoxicity, could be mixed with biodegradable polymers or treated with electrospun nanofiber scaffolds to make a substrate with the proper electrical conductivity. In one study, Golafshan et al. [132] investigated the potential of Gr nanosheet-sodium alginate/PVA fibrous scaffolds with exceptional toughness and electrical conductivity for neural tissue engineering. The addition of a 1 wt% Gr nanosheet produced a critical decrease in the impedance value (18-fold), which they attributed to the generation of conductive networks by the Gr. In another study, an aligned polypyrrole (PPy)/Gr/PLGA nanofibrous scaffold was used as the electrode to stimulate the growth of retinal ganglion cells (RGCs). The PPy-Gr/PLGA nanofibers provided a proper 3D environment with a high surface area for cell adhesion, and their excellent conductivity suggested effective electron transport during the electrical stimulation, which caused a 137% enhancement in cell length, with an improved antiaging effect for RGCs. This study verified that both conductive polymers and electrical stimulation could be excellent candidates as scaffolds in the regeneration of nerves [133].

### Bone tissue engineering

Bone healing is a complicated process that involves a cascade of osteogenic events. Consequently, bone tissue engineering demands the fabrication of engineered tissues that use scaffolds, cells, and soluble or mechanical factors. Biomimetic scaffolds for bone tissue engineering can have the following aspects: reconstruction of the nanofibrous collagen in ECM, high porosity to facilitate cell ingrowth and differentiation, and resistance to mechanical stress during tissue neogenesis. Electrospun nanofibrous scaffolds for bone regeneration have been developed using bioactive inorganics, biodegradable polymers, and their composites, forming fibrous nanocomposites with compositions and construction akin to the basic building blocks of inherently mineralized collagen nanofibers [134]. Xie et al. [135] seeded induced pluripotent stem cell-derived mesenchymal stem cells (iPSC-MSCs) on HA/COL/chitosan (CS) composite nanofibers. They reported that runt-related transcription factor 2 (Runx2) expression in the HA/COL/CS scaffold was enhanced by 2.85-fold compared with that in the tissue culture plate (TCP). Moreover, the ALP activity increased 54.6% in respect to the TCP, showing the effectiveness of the HA/COL/CS in improving iPSC-MSC osteogenic differentiation.

Electrospun SF/PLCL nanofibrous scaffolds cultured with hADSCs were used for in vitro bone regeneration. The SF/PLCL (50/50) scaffold displayed desirable tensile strength (6 MPa). In addition, its higher ALP activity, with an absorbance index of 150 compared with pure PLCL (absorbance activity of 80), demonstrated the ability of SF to promote the osteogenic differentiation of hADSCs [136]. Bhattacharjee et al. [137] electrospun PCL doped with nano HA and grafted with SF into nanofibrous scaffolds for bone regeneration. The SF-PCL with a 25% concentration of nano HA not only possessed higher mechanical properties, with an ultimate tensile strength of 16.06 ± 0.64 MPa, but also enhanced ALP activity. In another study, Zhu et al. [138] claimed that incorporating adenoviral vector-human bone morphogenetic protein 2 into an electrospun PLGA nanofibrous scaffold favored the proliferation and adherence of bone marrow stromal cells due to its high porosity and relatively small fiber diameter (140 ± 40 nm). Moreover, Runx2, ALP, and collagen type I were highly expressed after 14 days of culturing. Electrospinning collagen that incorporates catecholamines and calcium can produce a scaffold that mimics the native bone structure and has good mechanical properties (Young’s modulus of 675.8 ± 81 MPa). In addition, the cellular ALP activity/cell number on day 14 was 0.12, whereas it was 0.075 in the TCP as a control group, demonstrating human fetal osteoblast cell differentiation in a collagen based scaffold [139]. Miszuk et al. [140] produced functionalized PCL electrospun scaffolds with HA and demonstrated enhanced ALP activity and osteogenic differentiation of mouse multipotent C2C12 cells due to presence of HA.

### Cartilage tissue engineering

Treating cartilage defects is a challenge for orthopedic surgeons because of the complexity of structure and function in articular cartilage tissue, which consists of extremely specialized chondrocytes that have different properties in different regions. From the superficial to the deep regions, the cartilage ECM proteins vary spatially, for example, as the proteoglycan and collagen type II concentrations increase and decrease, respectively. Many tissue engineering approaches have applied hydrogel scaffolds, collagen sponges, and gelatin-based microspheres to cartilage regeneration. Moreover, electrospun nanofibers made from natural, synthetic, and composite polymers have been investigated as well because they have a structure similar to that of the ECM in native cartilage [141]. Electrospun nanofibers can promote the stiffness of the matrix as well as the biological properties of scaffolds such as cell–matrix interaction and chondrogenic differentiation. A study by Li et al. [142] illustrated that electrospun PCL scaffolds with enormous surface area not only improved the cell–matrix interaction, but also enhanced the differentiation of chondrocytes without the need for any growth factor.

Aligned nanofibers can mimic the organization of collagen fibrils in the native ECM of cartilage, and many studies have examined the potential for scaffolds with aligned nanofibers to form the superficial region of articular cartilage. Baker et al. [143] prepared electrospun scaffolds with an aligned structure by using 60% and 40% of PCL and PEO fibers, respectively. The PEO fibers were used to create macropores by dissolving the structure. The enlarged pores accelerated the infiltration of the fibro-chondrocytes, and after 9 weeks, the cells had highly proliferated and thoroughly infiltrated through the scaffold. Wimpenny et al. [144] fabricated a graded electrospun scaffold using coated poly (l,d-lactic acid) (PLDLA) nanofiber on PLDLA microfibers to form aligned nanofibers. The micro-sized pores caused by the microfiber’s presence enhanced the chondrocyte migration and infiltration. Collagen type II and aggrecan, as chondrogenic markers, were highly expressed because the fiber alignment induced the chondrocyte morphology.

### Tendon/ligament tissue engineering

Tendon and ligament injuries, generally prevalent in physically active young people, present a significant clinical challenge because of their intrinsically poor healing capacity. Natural healing typically forms scar-like tissue that has poor mechanical properties. Thus, tissue engineering is a promising alternative approach to tendon and ligament regeneration. Electrospun nanofibrous scaffolds have been applied to the regeneration of ligaments and tendons by offering an artificial ECM that is similar to the collagen fiber bundles of the natural tissues [145]. Normal healthy tendons are mainly composed of parallel arrays of closely packed collagen fibers, which produces highly anisotropic mechanical properties. Accordingly, scaffolds made of aligned nanofibers are promising candidates for tendon and ligament tissue engineering because they mimic the anisotropic structure of the native tissues. Several studies have investigated the effects of nanofiber diameter and alignment on the cellular behavior of both undifferentiated stem cells and committed fibroblast [146, 147].

A 3D multilayered scaffold was prepared by Jiang et al. [148] by incorporating PCL-poly (ethylene glycol) nanofibrous mats into porous chitosan. They found that the depth of cell filtration in the 3D aligned nanofiber-embedded scaffold (3D-AL) was considerably higher than in the 3D random (3D-RD) and porous control scaffolds, 45 μm, 38 μm, and 25 μm, respectively. In addition, 7 days after seeding, the cell viability on the 3D-AL was considerably higher (OD value about 2.2) than on the 3D-RD scaffold (OD value about 1.5). In another study, Perikamana et al. [149] reported that immobilizing platelet-derived growth factor BB in a gradient, aligned nanofiber scaffold significantly enhanced the maximum expression of tenomodulin, from 18.50 ± 1.45 to 65.79 ± 6.07 at day 14 compared with a random nanofiber scaffold (18.50 ± 1.45). Taylor et al. [150] fabricated an electrospun PLGA nanofibrous scaffold and reported that after 8 weeks, the supraspinatus tendon augmented by the scaffold showed a higher Young’s modulus (48.6 MPa) than the supraspinatus tendon that received only primary repair (3.79 MPa) in an in vivo study. Orr et al. [151] also proposed an aligned multilayered electrospinning approach to improve the cell infiltration and collagen deposition through PCL scaffolds seeded with hADSCs. The fold change in collagen type III and tenomodulin increased from 2 to 2.5 and 3 to 25, respectively, compared with nonaligned multilayered scaffolds, implying improved tendon-related gene expression.

### Clinical perspectives of electrospun nanofibers

Even though electrospinning is known as a simple, low-cost, and versatile method to prepare fibrous scaffolds by nano-meter scale with enormous potential to create multifunctional materials used in tissue engineering, its clinical application has not yet been fully exploited in the market. Several companies have made significant technical progress in this field, but none of the products have yet been approved from the FDA [152, 153]. For instance, Nicast developed a vascular access graft, AVflo™, prepared from polycarbonate-urethane and silicone with multilayered electrospun configuration [154]. Zeus^®^ produced an electrospun PTFE graft named Bioweb™ with application in scaffolding, stent encapsulation, and implantable structures in the body [155]. St. Teresa Medical, Inc^®^ developed SURGICLOT^®^, used as a hemostatic dressing, in which electrospun fibers deliver proteins to promote blood clotting, however, not yet commercially available [156]. Besides issues associated with safety and efficacy of electrospun fibers, there remain economical and technical challenges that should be addressed to realize their clinical applications. From an economic point of view, electrospinning not only suffers from low productivity yield, but also requires highly-skilled employees to make and develop high-quality products. From a technical standpoint, lack of advanced and robust process and product quality control is a critical issue. For instance, large quantity production of commercial products from electrospinning set-up in a continuous process is still quite challenging. The enormous potential of electrospun nanofibers in tissue engineering may be realized and translated into clinical outcomes by addressing the aforementioned challenges [157].

## Conclusions and outlook

In this review, we have reported the current progress in using the electrospinning technique for nanofibrous scaffolds, focusing mainly on preparation methods, materials (synthetic, natural, and composite polymers), and surface-structural modifications. The processing parameters, including voltage, distance between the needle and collector, type of spinneret, and flow rate, can greatly affect the nanofiber assembly. Solution parameters, such as polymer concentration, solvent type, and conductivity, also affect the structural morphology of the fabricated nanofibers. Nanofibers with the desired features should not only have a porous structure and high specific surface area, but they should also mimic the intrinsic properties of the native in vivo microenvironment. The materials used for nanofiber fabrication are also of pivotal importance in the electrospinning process. Scaffolds fabricated with natural polymers mainly suffer from low mechanical strength and structural integrity. On other hand, synthetic polymers possess good mechanical properties, but they are not suitable for cell attachment due to their low biological compatibility. Therefore, many attempts have been made to design novel composite materials that combine the advantages of both types of aforementioned polymers. Blending synthetic polymers with natural ones and embedding inorganic nanoparticles and conductive materials have been extensively investigated to promote the biocompatibility of the scaffolds. Surface modification techniques, such as plasma treatment, PDA coating, and wet chemical treatments, have been used to tune the functionality of the nanofibrous scaffolds. Structural modification of the nanofibers also plays a role in the elongation, proliferation, and differentiation of cells. The ideal alignment of nanofibers is of great importance to mimic the structure of native tissues such as tendons, blood vessels, and nerves.

Although electrospun nanofibers have shown excellent potential in tissue engineering applications, numerous technical issues remain to be resolved. The vast majority of published studies have reported in vitro results. Therefore, polymeric nanofiber scaffolds still need further optimization of their composition and structure for in vivo applications. Future studies need to focus on creating 3D porous scaffolds combined with cells and growth factors to optimize the infiltration and viability of cells. Additionally, it is important to push electrospun nanofibers from the laboratory to industrial scale. In summary, although many challenges remain, electrospinning appears to be an attractive method for the fabrication of functional nanofibers, enabling researchers from different disciplines to design and produce novel substrates for tissue engineering with desirable targets.

## Data Availability

The datasets used and/or analyzed during the current study are available from the corresponding author on reasonable request.

## Abbreviations

AFs: aligned fibers
ALP: alkaline phosphatase
CaP: calcium phosphate
CNTs: carbon nanotubes
CMWCNT-g-PHB: carboxyl multi-walled carbon nanotube-grafted polyhydroxy butyrate
CNFs: cellulose nanofibrils
CS: chitosan
COL: collagen
MADO: dopamine meth acrylamide-co-methyl methacrylate
ECM: extracellular matrix
AuNPs: gold nanoparticles
Gr: graphene
hADSCs: human adipose stem cells
hMSCs: human mesenchymal stem cells
HA: hydroxyapatite
LN: laminin
LBL: layer by layer
tHA: treated-HA
PDA: poly dopamine
PCL: poly e-caprolactone
PCFSs: poly e-caprolactone/collagen composite fibrous scaffolds
PEUU: poly ester-urethane urea
PEI: polyethylene imine
PEO: polyethylene oxide
PGA: poly glycolic acid
PHB: poly hydroxy butyrate
PLA: poly lactic
PLLA: poly-l-lactide
PLCL: poly l-lactic acid-co-e-caprolactone
PLDLA: poly l,d-lactic acid
PLGA: poly lactide-co-glycolide
PPy: poly pyrrole
PVA: poly vinyl alcohol
PVDF: poly vinylidene fluoride
RFs: random fibers
RGCs: retinal ganglion cells
SF: silk fibroin
AgNPs: silver nanoparticles
TPU: thermoplastic polyurethane
3D: three dimensional
TCP: tissue culture plate
